# Fluoxetine does not enhance the effect of perceptual learning on visual function in adults with amblyopia

**DOI:** 10.1038/s41598-018-31169-z

**Published:** 2018-08-27

**Authors:** Henri J. Huttunen, J. Matias Palva, Laura Lindberg, Satu Palva, Ville Saarela, Elina Karvonen, Marja-Leena Latvala, Johanna Liinamaa, Sigrid Booms, Eero Castrén, Hannu Uusitalo

**Affiliations:** 1 Herantis Pharma Plc, 02600 Espoo, Finland; 2 Neuroscience Center, HiLIFE, University of Helsinki, 00014 Helsinki, Finland; 3 Department of Ophthalmology, Helsinki University Hospital, 00029 Helsinki, Finland; 4 PEDEGO Research Unit, University of Oulu, 90014 Oulu, Finland; 5 Oulu University Hospital and Medical Research Center, 90029 Oulu, Finland; 6 Department of Ophthalmology, University of Tampere, School of Medicine, 33014 Tampere, Finland; 7 Tays Eye Center, Tampere University Hospital, 33521 Tampere, Finland

## Abstract

Amblyopia is a common visual disorder that is treatable in childhood. However, therapies have limited efficacy in adult patients with amblyopia. Fluoxetine can reinstate early-life critical period-like neuronal plasticity and has been used to recover functional vision in adult rats with amblyopia. We conducted a Phase 2, randomized (fluoxetine vs. placebo), double-blind, multicenter clinical trial examined whether or not fluoxetine can improve visual acuity in amblyopic adults. This interventional trial included 42 participants diagnosed with moderate to severe amblyopia. Subjects were randomized to receive either 20 mg fluoxetine (n = 22) or placebo (n = 20). During the 10-week treatment period, all subjects performed daily computerized perceptual training and eye patching. At the primary endpoint, the mean treatment group difference in visual acuity improvement was only 0.027 logMAR units (95% CI: −0.057 to 0.110; p = 0.524). However, visual acuity had significantly improved from baseline to 10 weeks in both fluoxetine (−0.167 logMAR; 95% CI: −0.226 to −0.108; p < 0.001) and placebo (−0.194 logMAR; 95% CI: −0.254 to −0.133; p < 0.001) groups. While this study failed to provide evidence that fluoxetine enhances neuroplasticity, our data support other recent clinical studies suggesting that improvement of vision can be accomplished in adults with amblyopia.

## Introduction

Amblyopia is a condition in which the best-corrected visual acuity (BCVA) is impaired in one eye or, less frequently, both eyes, even though no ocular abnormalities are generally present. Amblyopia develops when one or both eyes have abnormal visual input (either physical or physiological) during the sensitive period in childhood (from birth to 6 years)^1,2^. Unilateral amblyopia is most commonly caused by strabismus, anisometropia, or both combined. In the clinical setting, amblyopia is often defined as a logarithm of the minimum angle of resolution (logMAR) BCVA of worse than 0.30 (Snellen equivalent: equal to or less than 20/40) and/or an interocular BCVA difference of 0.2 logMAR or more (in cases of unilateral amblyopia). The prevalence of amblyopia in the general population varies from 1.3% to 3.6%, and it is one of the most common causes of monocular visual impairment in adults^3–5^. Patients with monocular amblyopia have a significantly increased risk of visual impairment if vision in their “good” eye is lost as a result of trauma or disease^6^.

Amblyopia can be treated in early life^7–9^, but visual gains diminish in school-aged children because of a decline in visual system neuroplasticity and, possibly, treatment compliance^1,10–12^. Historically, amblyopia in adults has been considered difficult, if not impossible, to treat^13^.

An improved understanding of the neuronal mechanisms underlying amblyopia and adult brain neuroplasticity^14–16^ has led to the development of visual rehabilitation methods that can be used after the critical period^17–21^. Experimental models of amblyopia are based on the effects of monocular deprivation on the structure and function of the visual cortex^22,23^. Using an animal model, Espinosa and Stryker^23^ showed that the effects of amblyopia can be reversed during the critical period in early postnatal development, but not later in life. However, recent evidence indicates that environmental enrichment^24,25^ and pharmacological treatment^26^ can reactivate critical period-like plasticity in the visual cortex of adult rodents. In particular, fluoxetine, a selective serotonin reuptake inhibitor (SSRI), promotes neuroplasticity and neurogenesis^27^ and reactivates critical period-like plasticity in the rat visual cortex^15^.

An emerging body of literature suggests that vision improvements can be achieved with conventional therapies (e.g., occlusion therapy) in adolescents (i.e., older children and teenagers)^28–30^ and adults^17,18,31,32^ with amblyopia even though post-childhood amblyopia has historically been considered untreatable. In addition, catecholamine-based medical treatments can temporarily improve vision in human amblyopic patients, including adults^1,33,34^. Perceptual training^35^, dichoptic non-action and action videogame use^36^, and videogame use during patching^37^ can improve vision in the amblyopic eye and binocular vision in adults. This placebo-controlled study examined whether fluoxetine can enhance neuroplasticity and improve vision in adults with amblyopia. The treatment included eye patching and computerized perceptual training on a web-based system for all subjects.

## Results

### Study subjects

**Figure 1:**
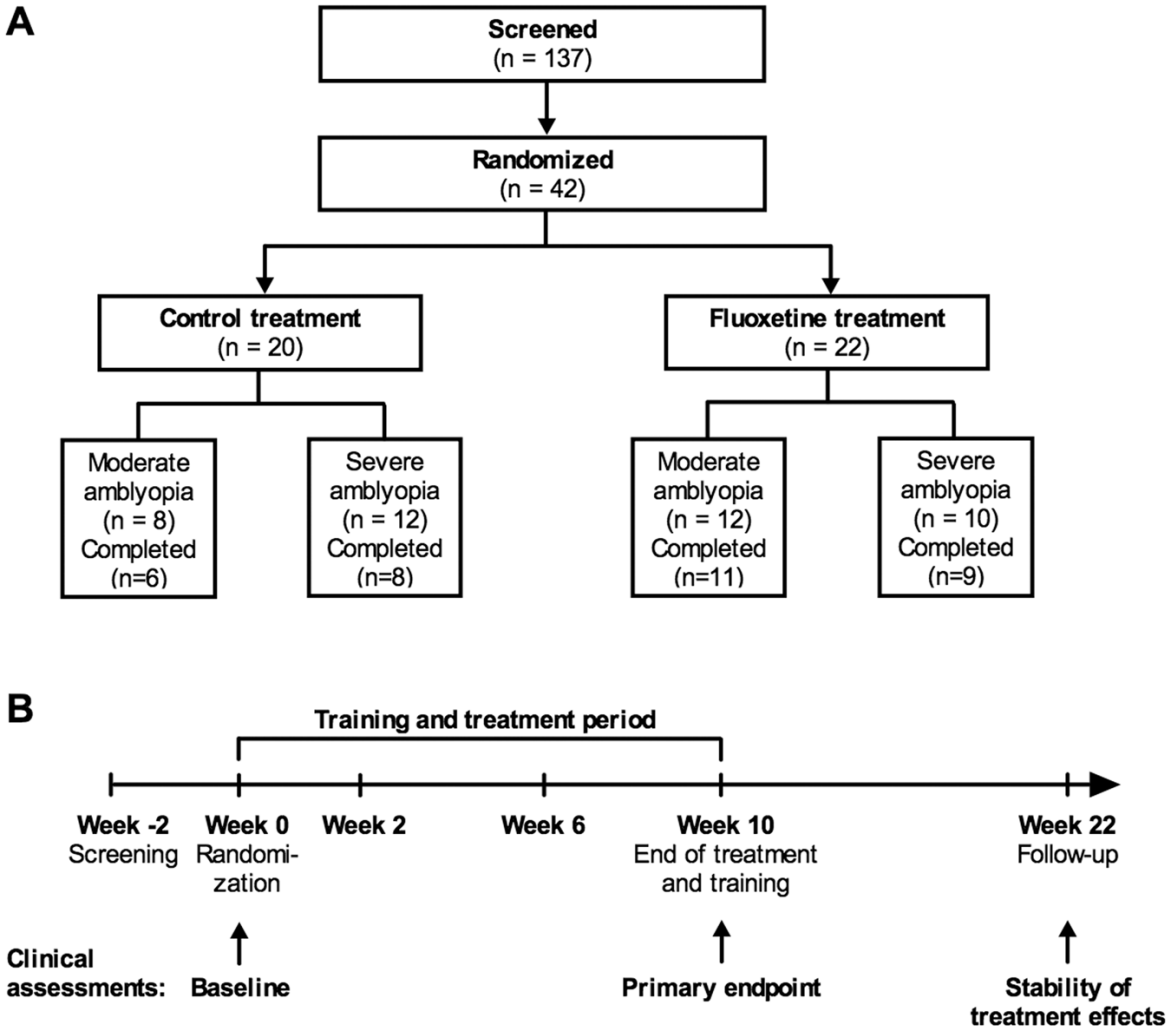
Study design and flow. (**A**) Disposition and study subjects. Subjects with moderate (0.3−0.6 logMAR interocular visual acuity difference) to severe (>0.6 logMAR difference) amblyopia were enrolled in the study. (**B**) Visit and assessment schedule and duration of medication and active training.

A total of 42 subjects were enrolled in the study, with 22 and 20 subjects randomly assigned to the fluoxetine and control group, respectively. Table 1 presents a complete list of the eligibility and exclusion criteria used for patient selection. Forty-one of 42 subjects (97.6%) required new spectacles before randomization. Four subjects were non-compliant, 3 subjects withdrew their consent, and 1 subject was lost to follow-up. Therefore, a total of 37 subjects ultimately completed the 10-week treatment period, including primary endpoint assessments, and 34 completed the 3-month post-treatment follow-up period (20 in the fluoxetine group and 14 in the control group; Fig. 1). Data from all 42 randomized subjects were subjected to an intention-to-treat analysis and were included in analyses. Subjects who completed the study showed good medication compliance (>85%) and completed >85% of the computerized perceptual training sessions.

**Table 1:** Eligibility and exclusion criteria.

**Eligibility criteria**
Age 18–60 years, male or femaleDiagnosed with amblyopia due to myopic or hyperopic anisometropia, or, congenital esotropia Visual acuity in the amblyopic eye ≥0.30 and <1.10 logMARVisual acuity in the dominant eye ≤0.10 logMARAnisometropia ≤4.25 (spherical equivalent in diopters)Judged to be otherwise healthy by the Investigator, based on medical history, brief physical examination, eye examination and clinical laboratory assessmentsFemales of childbearing potential were eligible for the study provided (i) they have a negative urine pregnancy test at the screening visit and (ii) they agreed to use adequate contraception (e.g. oral, depot or implanted hormonal contraception, intrauterine device, surgical sterilization or partner vasectomy) from the screening visit until at least 4 weeks after the last dose of study medication
**Exclusion criteria**
Diagnosed with other reasons of strabismus (than infantile esotropia) as the primary reason for amblyopiaHistory of any amblyopia therapy in the 2 years before the screening visitAny eye surgery less than 6 months before the screening visitObserved off-fixation by ophthalmological examination (extra-foveal/eccentric fixation)Other ophthalmological pathologies that may affect the patient’s rehabilitationPregnant, planning to become pregnant during the study, or breast feedingHistory of depressive illness or treatment with antidepressant medication within 6 months before the screening visitUse of psychiatric medication within 6 months before the screening visitReceipt of an experimental treatment for any disease within 4 weeks before the screening visitHistory or presence of illicit drug use or alcohol abuseHistory or presence of any medical or psychiatric condition or disease, or laboratory abnormality that, in the opinion of the Investigator, may place the patient at unacceptable risk or that could prevent the patient from completing the study

Baseline demographic and ocular parameters are summarized in Table 2. Briefly, best-corrected logMAR visual acuity at baseline was between 0.30 and 1.08 in the amblyopic eye and not worse than 0.10 in the dominant eye. The majority of subjects had anisometropic amblyopia [19 of 20 control subjects (95.0%), 18 of 22 fluoxetine subjects (81.8%)]. Four subjects [1 (5.0%) in the control group and 3 (13.6%) in the fluoxetine group] exhibited combined strabismic-anisometropic amblyopia, while 1 (4.5%) subject in fluoxetine group exhibited strabismic amblyopia. These 5 subjects had undergone strabismus surgery during childhood. Twenty subjects [8 (40.0%) in the control group and 12 (54.5%) in the fluoxetine group] had moderate amblyopia (0.30–0.60 logMAR) and 22 [12 (60.0%) in the control group and 10 (45.5%) in the fluoxetine group] had severe amblyopia (>0.60 logMAR)(Fig. 1A).

**Table 2:** Demographic data and mean visual parameters at baseline.

Demographic data and mean visual parameters at baseline		
	Control (n = 20)	Fluoxetine (n = 22)
Age (y)	36.4 ± 11.5 (19–57)	38.5 ± 12.5 (20–57)
Gender	11 male (55%)	11 male (50%)
	9 female (45%)	11 female (50%)
**Cause of amblyopia**		
Anisometropia	19 (95%)	18 (82%)
Anisometropia and strabismus	1 (5%)	3 (14%)
Strabismus only (congenital esotropia)	0 (0%)	1 (4%)
Mean visual acuity (logMAR, amblyopic eye)	0.620 (0.190)	0.649 (0.252)
Interocular visual acuity difference (logMAR)	0.728 (0.257)	0.728 (0.245)
>0.2 to <0.5	4 (20%)	6 (27%)
>0.5 to <0.8	7 (35%)	9 (41%)
>0.8	9 (45%)	7 (32%)
**Refractive error in amblyopic eye (D)** ^**1**^		
<0	1 (5%)	3 (14%)
≥0 to ≤+1.00	4 (20%)	3 (14%)
>+1.00 to <+3.00	4 (20%)	7 (32%)
≥+3.00	11 (55%)	9 (40%)
Mean (SD), hyperopes, n = 33	+3.13 (1.49)	+3.07 (1.79)
Mean (SD), myopes, n = 9	−1.23 (0.66)	−2.81 (1.07)
**Refractive error in non-amblyopic eye (D)** ^**1**^		
<0	4 (20%)	3 (14%)
≥0 to ≤+1.00	10 (50%)	10 (45%)
>+1.00 to <+3.00	4 (20%)	7 (32%)
≥+3.00	2 (10%)	2 (9%)
Mean (SD), hyperopes, n = 33	+1.43 (1.89)	+1.14 (1.04)
Mean (SD), myopes, n = 9	−0.95 (0.70)	−2.28 (1.20)
Mean anisometropia (D)^1^	2.19 (1.86)	1.95 (1.44)
Mean contrast sensitivity (log, amblyopic eye)	1.913 (0.096)	1.732 (0.568)
Crowded near visual acuity (decimal, amblyopic eye)	0.171 (0.116)	0.165 (0.141)
Binocularity (normal fusion in Bagolini striated glasses test, 33 cm)	13 (65%)	14 (64%)
Binocularity (normal fusion in Bagolini striated glasses test, 4 m)	11 (55%)	9 (41%)

^1^Spherical equivalent in diopters

Mean subject age was 36.4 ± 11.5 years in the control group and 38.5 ± 12.5 years in the fluoxetine group. Mean baseline logMAR visual acuity in the amblyopic eye was 0.620 ± 0.190 (Snellen equivalent: 20/83) in the control group and 0.649 ± 0.252 (20/89) in the fluoxetine group. The mean interocular difference in visual acuity between the amblyopic and fellow eyes was 0.728 in both groups. For hyperopic subjects (n = 33), the mean refractive error in the amblyopic eye was +3.13 ± 1.49 and +3.07 ± 1.79 D in the control and fluoxetine groups, respectively, and for myopic subjects (n = 9), the mean refractive error in the amblyopic eye was −1.23 ± 0.66 and −2.81 ± 1.07 D, respectively. Mean anisometropia was 2.19 ± 1.86 and 1.95 ± 1.44 D in the control and fluoxetine groups, respectively. Baseline binocular vision testing revealed that 9 of 20 (45.0%) control group subjects and 13 of 22 (59.1%) fluoxetine group subjects had suppression or anomalous retinal correspondence (ARC) at the 4-m distance. At the 33-cm distance, 7 of 20 (35.0%) subjects in the control group and 8 of 22 (36.4%) subjects in the fluoxetine group showed suppression or ARC at baseline. All subjects had impaired near visual acuity (with crowding effect; near logMAR visual acuity <0.7) at baseline, but only 2 (10.0%) control group subjects and 6 (27.3%) fluoxetine group subjects had abnormal contrast sensitivity at the intermediate spatial frequencies measured using the Pelli-Robson chart. Mean baseline visual parameters are summarized in Table 2.

**Figure 2:**
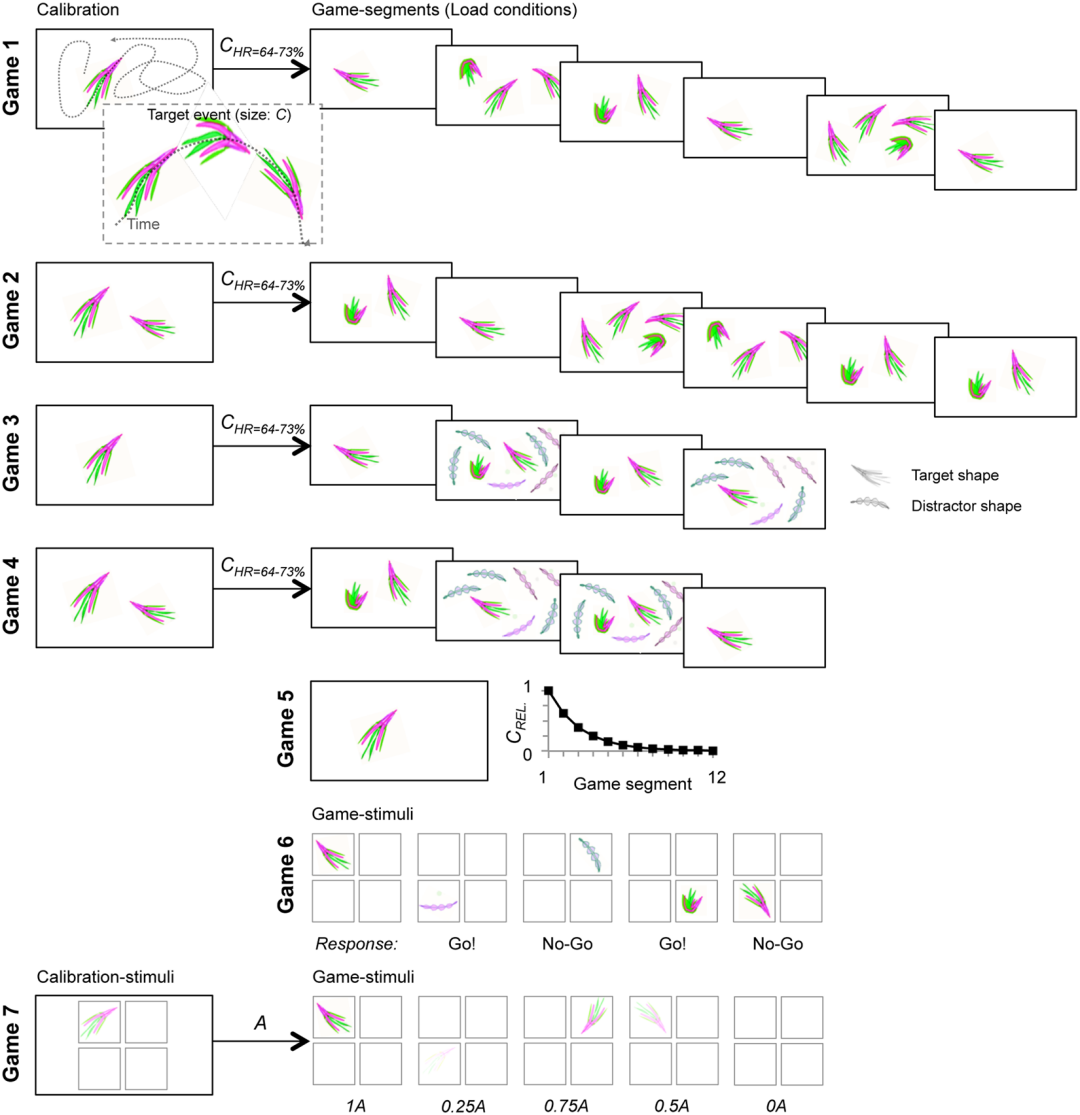
Schematic illustration of the training game task design and composition. For details, please see Materials and Methods section: *Training paradigm*. In short, the training program comprised seven different games, Games 1–7, that tapped primarily on visual acuity and contrast sensitivity in multiple attentional and working memory tasks. Subjects were presented with a pre-determined selection of games for each training day. The total training duration per week was ~3.5 h, excluding the time spent on game parameter adaptation. In all tasks, the subject responded with a single keyboard-button press or withheld the response. Games 1 and 2 were single- or multi-object visual tracking tasks where complex shaped objects moved along curved paths on screen and the subjects’ task was to respond whenever they observed a feature-change in any of the objects. Different game segments exhibited different numbers of to-be attended objects (attentional loads 1, 2, 3, and 4). Prior to each game, there was a calibration period with one (Game 1) or two (Game 2) objects during which the magnitude of the feature change (*C*) was adjusted to yield a detection rate (HR) of 64−73%. Games 3 and 4 were visual-tracking games like Games 1 and 2 and had an identical calibration procedure and object mobility, but involved only attentional loads of 1 and 2, and exhibited in two out of four conditions six feature-wise distinct distractor objects to impose visual crowding. Game 5 was a continuous single-object tracking task where the subjects reported the feature changes of a single object (as in Games 1−4). Game 5 had no calibration but rather started with very salient feature changes that in each of the 12 game segments decreased by a factor of 1.6 so that the subjects on average were able to reach segments 7−8 at a detection rate of >25%. Game 6 was a Go/No-Go 1-back working memory task where the subject was presented stimuli with an object in one quadrant lasting ~1 s at a rate of one stimulus in ~2.5 s. The subjects task was to indicate whether the object in the current stimulus was different from the one in the previous stimulus regardless of quadrant and object rotation. Game 7 was a threshold-stimulus-detection task where semi-transparent complex visual objects were presented randomly for 0.1 s and the subjects’ task was to report perceived stimuli. The object transparency was calibrated so that for an alpha-level A, detection rate of 0.5 was obtained at 0.5 A. During the games, objects were at five equiprobable levels of A so that A were 0, 0.25, 0.5, 0.75, and 1.0.

The 10-week treatment regimen included a combination of medication, refractive correction, eye patching, and perceptual training. A game-based perceptual training software was specifically developed for enhancing the use of the amblyopic eye during patching. The game tasks are illustrated in Fig. 2 and Supplementary video 1. The study was designed to include a placebo control group for the medication only, and all participating subjects were prescribed the same daily patching and training instructions.

### Visual acuity

**Figure 3:**
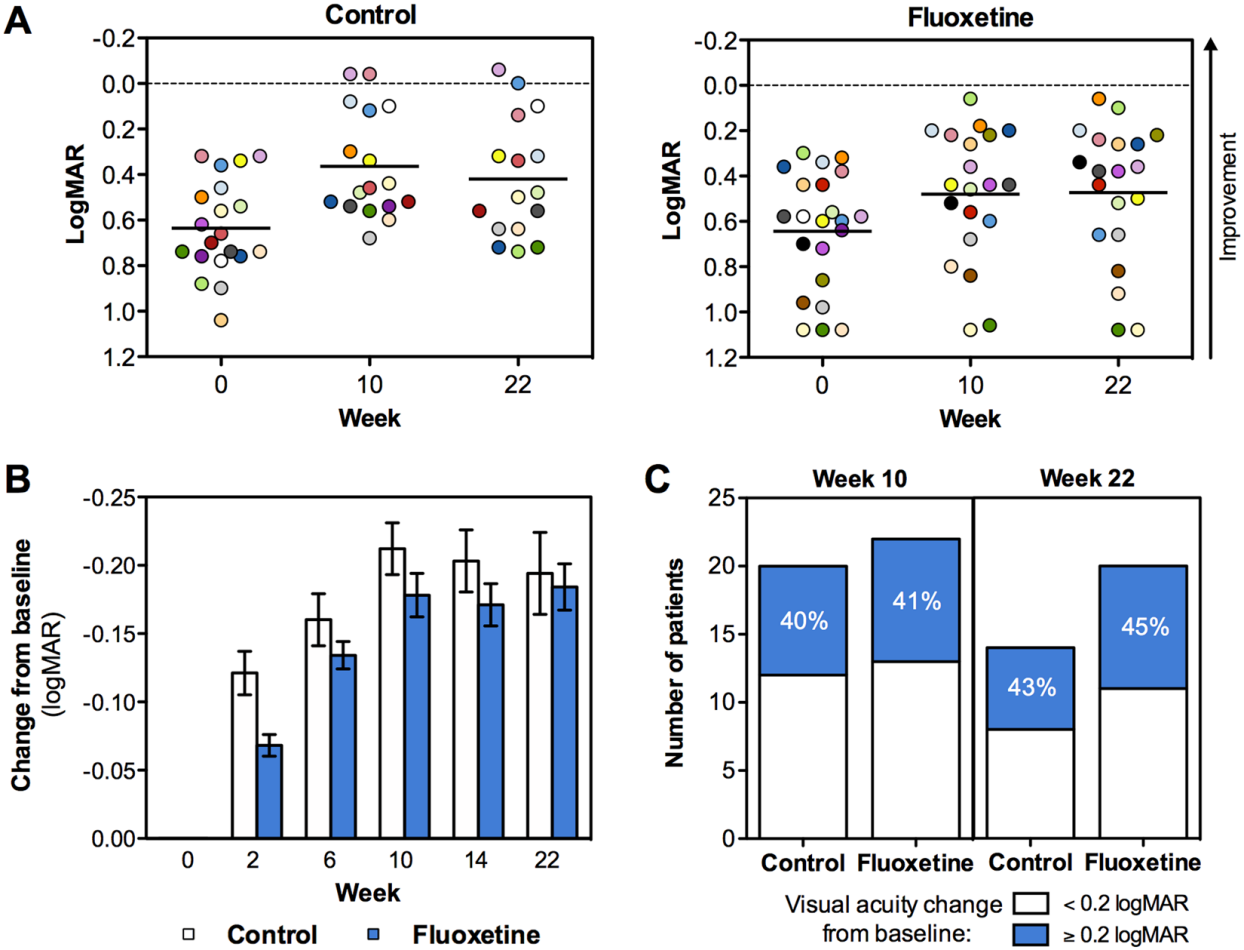
Improvement of visual acuity. (**A**) Scatter plots showing each individual patient’s visual acuity (amblyopic eye) at baseline, at week 10 (end of treatment/training) and at week 22 (end of follow-up), as measured by ETDRS chart (logMAR). Control group is shown on the left and fluoxetine group on the right. The limit of normal visual acuity (logMAR 0) is shown with a hatched line. (**B**) Average change in visual acuity from baseline as measured by ETDRS chart (logMAR) at baseline and after 2, 6, 10, 14 and 22 weeks. Average +/− 95% CI in each timepoint when visual acuity was determined by ETDRS chart is shown. (**C**) Number of patients per group who showed improved visual acuity by ≥0.2 or <0.2 logMAR units at week 10 and 22 as compared to baseline.

Visual acuity significantly improved in the amblyopic eye in both treatment groups (Fig. 3A,B). At the primary efficacy endpoint (10 weeks), the change in logMAR visual acuity from baseline was −0.167 [95% confidence interval (CI): −0.226 to −0.108; *p* < 0.001] in the fluoxetine group and −0.194 (95% CI: −0.254 to −0.133; *p* < 0.001) in the control group (Fig. 3A,B and Table 3). The mean treatment group difference in visual acuity improvement was only 0.027 logMAR units (95% CI: −0.057 to 0.110; *p* = 0.524). Nine subjects (42.8%) in the fluoxetine group and eight subjects (40.0%) in the control group improved visual acuity ≥0.2 logMAR units at the primary efficacy endpoint (Fig. 3C). Two subjects (9.5%) in the fluoxetine group and two subjects (10.0%) in the control group had improved to normal visual acuity ( < 0.10 logMAR) by the end of the 10-week treatment period.

**Table 3:** Summary of primary and secondary outcomes (from baseline to 10 weeks).

Primary endpoint: visual acuity (logMAR)							
	Mean change from baseline	95% CI	p^1^	Number of subjects with ≥0.2 logMAR improvement	Mean treatment group difference	95% CI	p^2^
Control	−0.194	−0.254 to −0.133	<0.001	8 (40.0%)	0.027	−0.057 to 0.110	0.524
Fluoxetine	−0.167	−0.226 to −0.108	<0.001	9 (42.8%)			
**Secondary endpoint: binocular vision (Bagolini striated glass test)**							
	**Number of subjects with improved binocular vision**			**p** ^**2**^			
Control	4 (23.5%)			p = 0.478			
Fluoxetine	2 (10.0%)						
**Secondary endpoint: contrast sensitivity (Pelli-Robson chart)**							
	**Number of subjects with improved contrast sensitivity**			**p** ^**3**^			
Control	0 (0%)			N/A			
Fluoxetine	2 (10.0%)						
**Secondary endpoint: crowded near visual acuity (Landolt C ring chart)**							
	**Mean change from baseline**		**95% CI**	**p** ^**1**^	**p** ^**2**^		
Control	0.181		0.126 to 0.236	<0.001	p = 0.381		
Fluoxetine	0.148		0.095 to 0.201	<0.001			

^1^Change from baseline to 10 weeks.

^2^Treatment group difference at 10 weeks.

^3^Contrast sensitivity was not analyzed statistically as there was practically no variation in the control group. Only two patients in the whole patient population had significant contrast sensitivity impairment at baseline. Both were in the fluoxetine group and both improved to almost normal contrast sensitivity from baseline to 10 weeks.

The visual acuity improvements observed at the 10-week primary outcome visit were maintained at the 22-week follow-up visit in both groups (12 weeks after treatment was discontinued at the primary outcome visit). Moreover, visual gains of at least 0.2 logMAR units persisted in many subjects in both groups [9 of 20 fluoxetine subjects (45.0%), 6 of 14 control subjects (42.9%); Fig. 3C]. The mean visual acuity in the control group changed from 0.636 (logMAR; 95% CI: 0.539 to 0.733) at the baseline to 0.365 (95% CI: 0.245 to 0.485) at 10 weeks, and to 0.420 (95% CI: 0.280 to 0.561) at 22 weeks. In the fluoxetine group, the mean visual acuity changed from 0.645 (logMAR; 95% CI: 0.530 to 0.760) at the baseline to 0.481 (95% CI: 0.345 to 0.618) at 10 weeks, and to 0.474 (95% CI: 0.331 to 0.617) at 22 weeks.

### Binocularity, contrast sensitivity and crowded near visual acuity

**Figure 4:**
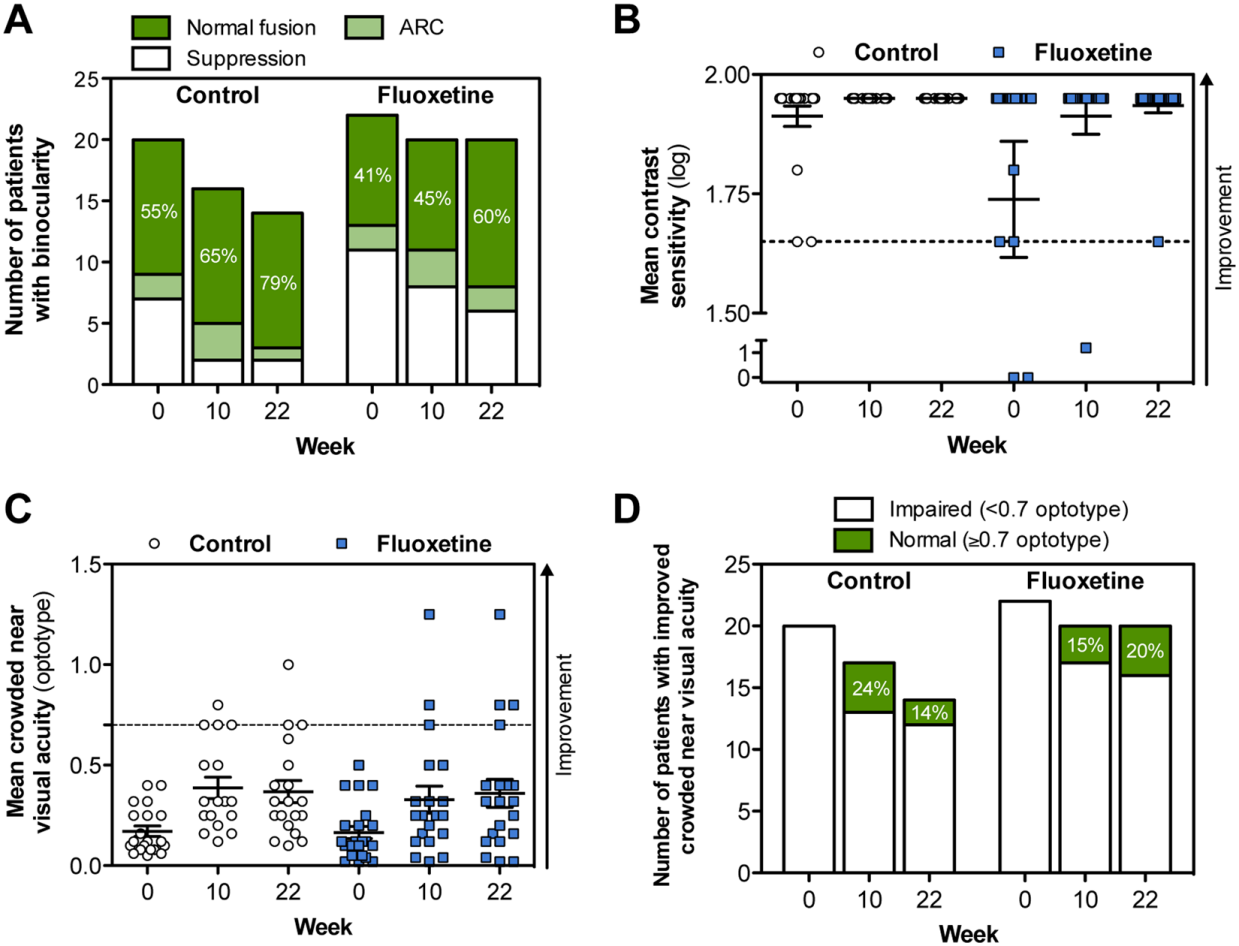
Change in binocularity, contrast sensitivity, crowded near visual acuity. (**A**) Number of patients per group who showed change in binocular vision (suppression, anomalous retinal correspondence (ARC) or normal fusion) at week 10 and 22 as compared to baseline. Bagolini striated glass test results at 4 meter distance are shown. (**B**) Mean contrast sensitivity (log value) as measured by Pelli-Robson chart. Normal contrast sensitivity (1.70) is indicated by a hatched line. Only two patients in the whole patient population (n = 42) had significant contrast sensitivity impairment (i.e. 0 log) at baseline. Both patients received fluoxetine and improved to almost normal contrast sensitivity. (**C**) Crowded near visual acuity as measured by Landolt C ring charts. Normal crowded near visual acuity was considered as ≥0.7 (hatched line). In panels B and C, the direction of improvement is indicated by an arrow. (**D**) Number of patients per group who improved in crowded near visual acuity test at week 10 and 22 as compared to baseline.

Similarly to visual acuity, improvements in binocular vision, contrast sensitivity, and crowded near visual acuity were observed in both the control and fluoxetine groups. Binocular vision was assessed for both near (33 cm) and distant (4 m) vision. Twenty-two subjects [9 (45.0%) control group subjects, 13 (59.0%) fluoxetine group subjects] had suppression or ARC in the 4-m test at baseline (Fig. 4A). At 10 weeks, the number of patients with suppression or ARC had decreased to 16 subjects [5 (31.3%) control group subjects, 11 (55.0%) fluoxetine group subjects]. This change persisted through 22 weeks in 11 subjects [3 (21.4%) control group subjects, 8 (40.0%) fluoxetine group subjects]. The results in the 33-cm test were similar for both groups (data not shown).

Contrast sensitivity was normal (≥1.70 log) at baseline in all but two (4.8%) fluoxetine group subjects who both had severe amblyopia (both had 0 log values for contrast sensitivity at the baseline). Following fluoxetine treatment and perceptual training, both subjects had improved to almost normal contrast sensitivity (at 10 weeks: 1.20 and 1.95 log, at 22 weeks: 1.95 and 1.65 log; Fig. 4B).

Crowded near visual acuity was assessed using Landolt C ring charts and was considered to be normal when 0.7 or smaller optotypes were detected. The baseline mean crowded near visual acuity in the amblyopic eye was 0.171 ± 0.116 in the control group and 0.165 ± 0.141 in the fluoxetine group. Improvements in these values were observed after treatment in both study groups (Fig. 4C). At the primary endpoint (10 weeks), the control group had improved by 0.181 ± 0.027 (95% CI: 0.126 to 0.236) and the fluoxetine group had improved by 0.148 ± 0.026 (95% CI: 0.095 to 0.201, both p < 0.001). At 22 weeks, the control group had improved by 0.221 ± 0.036 (95% CI: 0.150 to 0.293) and the fluoxetine group had improved by 0.197 ± 0.033 (95% CI: 0.131 to 0.262, both p < 0.001). Figure 4D shows the distribution of patients with normal (≥ 0.7) and impaired (<0.7) crowded near visual acuity at the baseline, and at 10-week and 22-week timepoints. Primary and secondary outcome data at the primary efficacy endpoint (10 weeks) is summarized in Table 3.

### Safety

A total of 66 adverse events (AEs) were reported after initiating study treatments. Fifty-eight (87.9%) AEs occurred during treatment and 8 AEs occurred (12.1%) after treatment. Only 16 AEs (24.2%) were related or possibly related to the study treatment and all were reported during the treatment period. Eleven (16.7%) of these AEs occurred in the fluoxetine group and 5 (7.6%) occurred in the placebo group. None of the treatment-related AEs were reported following the 10-week treatment period and no AEs led to study withdrawal. One subject in the fluoxetine group exhibited transient mild diplopia that resolved spontaneously. Other reported AEs were not related to visual function. One serious adverse event (benign ovarian cyst of moderate severity) occurred during the study, but it was not related to study treatment.

## Discussion

Amblyopia is a complex brain disorder that can restrict everyday life because of the visual limitations it imposes. Despite good screening programs and effective childhood treatments, amblyopia remains a common cause of lifelong visual impairment independent of geographical location or ethnic origin^3,5,6,38^. The combination of adequate refractive correction and occlusion therapy (patching of non-amblyopic eye) has been the mainstay therapy for amblyopia of all etiologies. However, the benefits of various forms of occlusion therapy are greatest when therapy is started at an early age (<8 years). Therefore, early amblyopia detection and treatment is the most important factor for obtaining successful visual outcomes. Physiologically, the brain has the greatest plasticity during the critical period in early postnatal life. However, recent evidence strongly indicates that the primary sensory cortex may remain plastic into adulthood^14–21,24–27^. This finding suggests that there is a physiological basis for treating amblyopia in adulthood, which provides an opportunity to potentially alleviate this world-wide public health problem.

The current study examined whether fluoxetine, an SSRI known to modulate adult rat visual cortex plasticity^15^, can enhance the effects of patching/computer-based perceptual training combination therapy in adults with amblyopia. The treatment response was good in both the fluoxetine and placebo groups. However, the change in visual acuity after 10 weeks of study medication/perceptual training therapy was not significantly different between subjects taking fluoxetine and subjects taking a matching placebo. It may be that 20 mg of fluoxetine, the dose typically used to begin treatment of depression, was too small a dose to modulate neuroplasticity. It may also be that the training paradigm (new spectacles, patching, and computerized perceptual training) was so effective that the 20-mg dose of fluoxetine did not provide any additional benefit. Larger fluoxetine doses (up to 80 mg/day) are often used in depressed patients when the initial dose does not have the desired therapeutic effect. In addition, the 10-week treatment period may have been too short to maximize fluoxetine benefits and it is possible that a difference between treatment groups could have emerged after a longer treatment period.

Although there is evidence that some amblyopia treatments may be additive (optical correction in combination with patching or atropine)^29^, some reports have documented that all amblyopia treatment effects may not be additive. The effects of multiple amblyopia treatment paradigms were not synergistic in rodent models of amblyopia^39^. Furthermore, environmental enrichment and fluoxetine treatment have been shown to induce similar levels of amblyopia recovery in rodents^25^. Therefore, it is possible that perceptual training alone (with refractive correction) promotes the maximum amount visual cortex plasticity and that further treatments do not have additive benefits. The current study was not designed to determine this and future studies should include a group of subjects only treated with fluoxetine (no perceptual training). However, a rodent study found that fluoxetine alone had no effect on vision^15^. Furthermore, there are no prior reports of visual benefits from fluoxetine monotherapy in amblyopic patients, even though millions of patients, and presumably thousands of amblyopic patients, have used the medication over the past three decades. In addition, a recent placebo-controlled, double-blinded clinical study showed that 20 mg/day of fluoxetine for 19 days did not significantly affect visual perceptual learning in humans^40^. These prior studies support the theory that 20 mg/day of fluoxetine may not be a large enough dose to effectively modulate visual cortex plasticity in adult humans.

Subjects in the current study received new spectacles with the proper refractive correction at baseline. Initiating the use of appropriate prescription glasses can improve amblyopia in children^41^ and adults^42,43^. Therefore, the use of new corrective lenses may have added to the visual gains observed in the current study. Furthermore, test-retest variability should be taken into account; this was low in the present study because visual acuity was measured under the same conditions and in the same locations by the same observers. Moreover, regression to the mean must be taken into account; because our study was placebo-controlled, the regression to the mean was reduced because both groups most likely exhibited an equal tendency.

Subjects in this study had an average gain of approximately 2 lines of vision (0.2 logMAR), which was similar to improvements observed with other published vision training protocols^35,36^. Our results are also in agreement with those of Li *et al*.^37^, who found that 33% of adult amblyopic subjects had a substantial improvement in visual acuity following video game-based perceptual training. A recently published randomized clinical trial using a falling blocks video game with dichoptic contrast offset vs a placebo game (no dichoptic presentation) found only modest visual acuity gains (<0.1 logMAR) in older children, teenagers and adults with amblyopia^44^. Notably, in this study, compliance requirements to prescribed game play were rather low (>25% of minimum prescribed dose or at least 10.5 hours at 6 weeks) which may have affected the results. In our study, the subjects completed >85% of the computerized perceptual training sessions (at least 29 hours during the 10-week treatment period), suggesting that the intensity of training may correlate with the magnitude of vision improvement.

Our study had several limitations. First, we did not have a true no-treatment control group. Future clinical trials combining multiple interventions should include several control groups to examine the effects of the individual interventions and their combined effects. Furthermore, the dose of fluoxetine and duration of its use should be varied in future studies. Second, the response to treatment was remarkably variable in both treatment groups. This may have resulted from the large amount of variation in amblyopia severity and etiology in our study population. Twenty-two subjects (52.4%) had abnormal binocularity at baseline, 10 of which had improvements in binocularity with study treatment. In addition, only 2 subjects, both with severe amblyopia, had low contrast sensitivity at baseline. Contrast sensitivity deficits are sometimes found to correlate with the visual acuity in the amblyopic eye^45–47^. Both subjects exhibited remarkable improvements in visual test results. Our results are in agreement with those of Zhou *et al*.^48^, who showed that perceptual learning can improve visual acuity and contrast sensitivity in adult amblyopia patients. Nine of our subjects (21.4%) had improved crowded near visual acuity. Hussain *et al*.^49^. found a significant association between reduction in crowding and visual acuity improvement in amblyopic adults. However, we did not observe a correlation between visual acuity improvement and crowding, contrast sensitivity, or binocularity.

In conclusion, both fluoxetine and software-based perceptual training were safe and well-tolerated, with fluoxetine treatment not offering further benefits over perceptual training. The software-based training tool developed for this study was found to be useful for following up training compliance and could be used for personalizing visual training in the clinical setting in the future.

## Materials and Methods

All study conduct adhered to the tenets of the Declaration of Helsinki and followed Good Clinical Practices. This study was reviewed and approved by the Regional Ethics Committee of Tampere University Hospital (centralized process for all centers in Finland) and the Research Ethics Committee of the University of Tartu (Estonia). Written informed consent was obtained from all subjects prior to performing any study examination or procedure. The study was registered in the European Clinical Trials Database (EudraCT) on October 1st, 2010 under the number 2010-023216-14.

This Phase 2, multi-center, clinical study was a randomized, double-blind, placebo-controlled (for drug treatment), parallel-group trial performed to assess visual acuity improvement in the amblyopic eye, as measured by the Early Treatment of Diabetic Retinopathy Study (ETDRS) chart, following 10 weeks of medication (20 mg fluoxetine or placebo) and computer-based training (with the dominant eye patched). The following assumptions were made to calculate sample size: comparison of two equally sized groups, an intergroup difference (fluoxetine vs. placebo) in the change in logMAR visual acuity of at least 0.15, a standard deviation (SD) of 0.15, and a subject drop-out rate of 10%. Thirty-four subjects needed to be randomized to power the study to 80%, assuming a two-sided type I error rate of 5%.

### Study subjects

Four eye clinics in Finland and Estonia enrolled 42 subjects between June 2011 and April 2013. Study inclusion and exclusion criteria are fully described in Table 1. Briefly, adult patients with monocular amblyopia with no other ocular or neurological abnormalities were considered for enrollment. Included subjects were 19 to 57 years of age and had moderate (0.3−0.6 logMAR difference) to severe (>0.6 logMAR difference) amblyopia due to myopic or hyperopic anisometropia (≤4.25 D) or congenital esotropia. The lower limit for anisometropia was not set in the study protocol. The investigators considered the amblyopia to be of the anisometropic type if no strabismus had been diagnosed in childhood and the refractive error was at least 1 D of anisometropia, determined as the spherical equivalent, in childhood. Patients with other primary forms of strabismus, extrafoveal (eccentric) fixation, or who used antidepressant drugs in the past 6 months were excluded.

### Study examinations

Eligibility, demographic data, medical history, relevant medication, vital signs, physical examination, blood and urine samples (including urine pregnancy test for fertile women), and amblyopia were assessed at screening. Amblyopia was confirmed at screening and was defined as an interocular ETDRS best-corrected visual acuity difference of at least two lines and/or a logMAR visual acuity between 0.30 and 1.10 in the amblyopic eye and 0.10 or better in the dominant eye. Prior to randomization, patients received new spectacles based on non-cycloplegic refraction to ensure best-corrected vision during the study.

A thorough ophthalmic examination was conducted at each of the seven scheduled visits (at weeks −2 (screening), 0 (randomization), 2, 6, 10, 14 and 22; Fig. 1) during the 26-week study period. Vision tests included the assessment of binocularity, visual acuity, crowded near visual acuity, and contrast sensitivity, and were performed with the refractive error corrected. In addition, presbyopic correction was used for crowded near visual acuity testing in presbyopic subjects.

Binocularity was examined using the Bagolini striated glass test^50^ before monocular testing. Lens striations were placed at 135° before the right eye and 45° before the left eye using lorgnette frames. This testing setup allows each eye to receive the same fusible image with each fixation streak oriented perpendicular to the striations and 90° away from the other eye. The test enables the evaluation of simultaneously perceived images with a minimal dissociative effect and it was performed at near (33 cm) and distance (4 m) under normal lighting conditions. Binocularity was categorized as suppression (1 light and only 1 line were seen), normal fusion (binocular single vision, BSV; 2 lines were seen as X and 1 light at the center), anomalous retinal correspondence (ARC; harmonious if 1 light and 2 lines were seen, but one of the lines was broken due to foveal suppression, or inharmonious if 1 light and 2 lines were seen, but the lines did not cross at the center where the light was located) or diplopia (2 lights and 2 lines were seen). None of the subjects had diplopia in the current study.

Visual acuity was assessed under standardized lighting conditions (self-calibrated test lighting with a constant light level of 85 cd/m^2^) using a large-format standardized ETDRS light box (ESV3000 with LED lights, VectorVision, Greenville, OH) placed 4 meters from the subject. Three different ETDRS charts (charts R, 1 and 2) were used to prevent subjects from memorizing eye charts. Visual acuity was assessed in the amblyopic eye first and was measured as the number of correctly identified letters. A clinically relevant visual acuity improvement was defined as a 0.2 or greater decrease in the logarithm of the minimum angle of resolution (logMAR) visual acuity (i.e., 2 lines or 10 characters on the ETDRS chart).

Contrast sensitivity was determined under standardized lighting conditions using a Pelli-Robson chart at a distance of 1 m (charts A and B), using previously established age-dependent normative values^51^. Crowded near visual acuity was assessed using a specific crowded Landolt C ring chart booklet at a distance of 40 cm^52^. Crowded near visual acuity was defined by the smallest line in which the subject correctly identified at least 8 of 12 letters (≥66.7%). The right eye was tested first in all tests requiring charts. The contralateral eye was occluded during testing and charts were switched between eyes. All eye examination test charts and Bagolini striated glasses tests were standardized and validated for trial endpoint measurement. All staff involved in vision testing were masked to subject group assignment.

Treatment safety was assessed using ophthalmoscopy, biomicroscopy, intraocular pressure (IOP) measurement, laboratory safety tests [hematology (hemoglobin, hematocrit, erythrocyte count, leukocyte count, platelet count), clinical chemistry (alanine aminotransferase, alkaline phosphatase, aspartate aminotransferase, creatinine, gamma-glutamyl transferase, potassium, sodium, urea), urine analysis (blood, glucose, ketones, protein, pH)], vital signs, and physical examination performed at screening and at each study visit. Adverse events and changes in concomitant medications were recorded at each study visit.

### Study medication

Fluoxetine capsules were manufactured by Orion Corporation (Espoo, Finland) and the matching placebo capsules were manufactured by Corden Pharma GmbH (Plankstadt, Germany). Study subjects were randomly assigned to receive either 20 mg fluoxetine each day (hard capsule) or a matching placebo. Randomization was done in a 1:1 fashion in blocks of 4 and was stratified by site. Randomization was also stratified by amblyopia severity, determined using interocular visual acuity difference (moderate: 0.3−0.6 logMAR difference, severe: > 0.6 logMAR difference). The randomization structure was designed by a biostatistician and the final randomization list was generated by an independent person who had no contact with study subjects or study data. Medication was pre-packed and serially numbered so that subjects were assigned to a study group by giving them the next available medication number in the sequence. A drug accountability log was maintained by study-authorized personnel. The receipt, dispense and return of study medication was recorded in this log. Patients were instructed to return dispensed medication bottles at the next visit, even if the bottles were empty. The number of capsules dispensed and returned was reconciled against the number of days between the visits and any discrepancies were accounted for. After 10 weeks of receiving study medication, subjects were weaned off the daily medication (1 capsule every other day for the next 2 weeks, Fig. 1B).

### Perceptual training

All subjects were prescribed daily computerized training with eye patching during the 10-week period of receiving study medication (Fig. 1A). The principle underlying the perceptual training software developed for this study is fully described in the Electronic Supplementary Materials and is illustrated in Fig. 2 and Supplementary video 1. All subjects received new spectacles before randomization and were instructed to wear an eye patch over their dominant eye while performing daily computerized perceptual training. The training software was used to track training compliance, which was calculated by dividing the total accomplished training time with the total prescribed training time. Training compliance was automatically reported to the study site prior to each scheduled visit.

All subjects were given an eye patch and were instructed to wear it over the non-amblyopic eye for 1 hour each day. Subjects were also instructed to complete approximately 30 minutes of the computer-based training each day while they were wearing the patch and their spectacles.

The training period was divided into ten 1-week segments and each subject played an identical composition of games each week. The maximum total training time over the 10-week training period was 35 hours. The training program was made up of seven different games wherein the performance was primarily determined by visual acuity and contrast sensitivity and secondarily by attention and mental effort. Thus, the training was primarily focused on visual acuity and contrast sensitivity and was aimed at their improvement. A schematic illustration of the training game task design and composition is shown in Fig. 2. In addition, the computerized training setup, training protocol structure, and individual training game design are described in detail in the Electronic Supplementary Materials. Data on behavioral performance were collected on a per-game basis. For each game type, the corresponding weekly test outcome measures were obtained by pooling the data from all individual games of that type played in that week (see Electronic Supplementary Materials).

### Study outcomes

The primary outcome of the study was an improvement in visual acuity in the amblyopic eye, as measured by the ETDRS chart, from baseline (week 0, randomization) to the 10-week visit (end of treatment). Secondary outcomes included the change from baseline in binocularity, contrast sensitivity and crowded near visual acuity to week 10 week. The persistence of changes observed at 10 weeks was also evaluated at the end of the follow-up period (week 22). Treatment safety was assessed using adverse event incidence and ophthalmological examination findings throughout the study. Exploratory outcomes included changes from baseline in training measures at each study visit.

### Data analyses

The primary analysis population was the full analysis dataset (FAS), which included all randomized patients who had received at least one dose of study medication (intention-to-treat principle). A last observation carried forward (LOCF) imputation was applied up to week 10 for subjects who did not complete the study and those who were non-compliant with the treatment.

Differences in visual acuity and crowded near visual acuity between the two treatment groups were evaluated using the repeated measurements of analysis of covariance (RM ANCOVA) method with baseline values as a covariate. The model included the study center, treatment and time point (visit) as main effects, and treatment by time point (visit) as an interaction effect. With regard to the primary endpoint, differences between the treatment groups with regards to change in the logMAR visual acuity at 10 weeks (and a 95% CI for the difference) were estimated using RM ANCOVA models with a contrast. A secondary RM ANCOVA analysis was used to compare the least square means between the treatment groups at the end of the follow-up (22 weeks) period to determine if treatment effects were maintained. Contrast sensitivity was not analyzed with RM models because of low variability in the dataset. Pearson’s chi-square test was used to compare categorical variables. Some binocularity categories contained a low number of subjects. Therefore, differences between treatment groups in binocularity were evaluated using Fisher’s exact test.

All statistical analyses were performed using SAS software version 9.3 (SAS Institute Inc., Cary, NC, USA). P values of less than 0.05 were considered to indicate statistical significance.

## Electronic supplementary material

Supplementary information

Supplementary video 1

CONSORT 2010 Checklist
