# Supplementary material for: Fluoxetine does not enhance the effect of perceptual learning on visual function in adults with amblyopia
Source: Sci Rep. 2018 Aug 27;8:12830. doi: 10.1038/s41598-018-31169-z (PMC6110780; doi:10.1038/s41598-018-31169-z)
Supplement: Supplementary file 1 — Supplementary information [file 41598_2018_31169_MOESM1_ESM.docx]

**Fluoxetine does not enhance the effect of perceptual learning on visual function in adults with amblyopia**

Henri J. Huttunen^1,2,*^, J. Matias Palva^2,*^, Laura Lindberg^3^, Satu Palva^2^, Ville Saarela^4,5^, Elina Karvonen^4,5^, Marja-Leena Latvala^6^, Johanna Liinamaa^4,5^, Sigrid Booms^1^, Eero Castrén^2^ and Hannu Uusitalo^6,7^

^1^Herantis Pharma Plc, 00790 Helsinki, Finland;

^2^Neuroscience Center, University of Helsinki, 00014 Helsinki, Finland;

^3^Department of Ophthalmology, Helsinki University Hospital, 00029 Helsinki, Finland;

^4^PEDEGO Research Unit, University of Oulu, 90014 Oulu, Finland;

^5^Oulu University Hospital and Medical Research Center, 90029 Oulu, Finland;

^6^Department of Ophthalmology, University of Tampere, School of Medicine, 33014 Tampere, Finland;

^7^Tays Eye Center, Tampere University Hospital, 33521 Tampere, Finland.

^*^ Equal contribution

**SUPPLEMENTARY VIDEO LEGENDS**

**Supplementary video 1.** Schematic video representation of the change detection task (Tasks 1 and 2). Green stars in lower left corner indicate successful responses while red crosses denote false alarms. The calibration phase ends when 7-8 successful responses was obtained in a block of 11 change detections. If less was found, the change magnitude was increased and if more, it was decreased (see Supplementary methods).

**SUPPLEMENTARY METHODS**

***Computerized training setup***

The subjects were instructed to cover their dominant eye and to play a series of visual games every day for an effective gaming time of approximately 30 minutes per day throughout the 10-week treatment period. The training thus comprised 70 days of training and the total training time at 100% compliance was 35 hours. Each subject was given an identical training paradigm where the total training period was divided into ten one-week segments so that each week comprised an identical composition of games.

All training was carried out on pre-configured laptop computers (HP ProBook 4525s) and through a mobile internet connection, both provided by the study sponsor. The computers had a 2.2 GHz CPU, ATI Mobility Radeon HD 4250 graphics processor and full-HD LED backlight 15.6” anti-glare displays, and were able to run the training program at around 60 frames per second, *i.e.*, near the screen refresh rate. The training software had an web browser-based user interface that allowed the participants to log in with their personal credentials, observe which games were due to be played on that day, launch and play these games, and inspect their game-performance history. After each game, the system gave feedback to the participant about the performance in that game and about how it compared against prior games. The user interface was programmed in-house in Flash (Adobe Systems). All game performance data storage, tracking of the participants’ progress in the experiment, determination of the daily games, and game-parameter adaptation to individual performance were performed through a web service in backend server software programmed in-house in LabVIEW (National Instruments).

The computers were locked from modifications so that they could not be used for other purposes than the training and that no computer settings, including display parameters such as resolution, refresh rate, or brightness, could be adjusted by the participant. At the Week 0 randomization visit, the participants were provided with the computers and individually instructed by the study staff on how to operate the computer, software user interface, and how to play each type of games. The participants were further instructed to operate the computer from an eye-to-monitor distance of approximately 80 cm, which yielded total screen dimensions of 24 x 13.5 visual degrees and a pixel density of 78.6 pixels per degree at center and ~80.4 at edge. The participants had access to dedicated technical support on all hard- and software-related issues throughout the trial. All participants thus received technically and visually similar training, and were competent in using the training system.

***Training protocol structure***

The training program comprised seven different games, Games 1−7, that were intended to strengthen visual acuity and contrast sensitivity in multiple cognitive task settings. Each training week comprised the following constant composition of games: Game 1, 4 x 4 games, total 24 min; Game 2, 4 x 4 games, total 24 min; Game 3, 4 x 3 games, total 24 min; Game 4, 4 x 3 games, total 24 min; Game 5, 4 x 3 games, total 28.8 min; Game 6, 4 x 4 games, total 48.8 min; Game 7, 4 x 3 games, total 36 min. Thus, the total training duration per week was ~3.5 h, excluding the time spent on game parameter adaptation.

The ten training weeks were distributed so that in the 1st, 3rd−5th, and 7th−9th weeks, adapting game parameters (see below) were unlocked and the games automatically adjusted to the participant’s individual momentary performance level in order maintain constant difficulty.

The 2nd, 6th, and 10th weeks were “assessment” weeks and corresponded temporally to the 3rd, 4th, and 5th ophthalmologist visits, respectively. During the assessment weeks, instead of the using the current adapting game parameters, the games were presented with parameter values locked to those obtained during the 1st week. Assessment week games thereby yielded estimates of the training-related change in task performance in a manner comparable between these weeks. Games 5 and 6 (see below) were non-adaptive and hence yielded comparable test outcome measures at each week of the experiment.

***Training games***

**Games 1 and 2** were multi-object tracking games where the subject tracked 1−4 complex moving objects (Figure 2) that moved on smoothly curved random paths on the full computer screen and exhibited irregular (uniform inter-event onset-to-onset range 0.6−2.4 s, mean 1.5 s) target events^1^. The subject’s task was to report the target events with a keyboard-button-press and the events were perceptually small and short-duration (0.12 s) feature changes in any the objects. Responses in less than 1.0 s from the target event were considered valid while responses after this limit were considered false alarms. If the subject produced more than six false alarms, the game was terminated and the participant had to restart it. The objects were 130 ± 26 pixels (1.65 ± 0.33 visual degrees) long and had ±50 % hue variability (Figure 2).

Prior to each game, the magnitude of the feature change was adjusted to yield a change-detection accuracy of ~0.7 when either one (Game 1) or two objects (Game 2) were attended. The adaptation was performed by presenting 11 consecutive target events and stopping the adaptation if 7 or 8 of them were detected. If <7 or >8 target events were detected, another 11 targets were presented with the feature-change magnitude increased or decreased, respectively, by 10 %. The final in-game accuracy in the corresponding attentional loads was 0.75 ± 0.03 (mean ± SD across all weeks and subjects) in both Game 1 and 2. Games 1 and 2 were thus adapted to the change-detection accuracy of foveal and peripheral vision, respectively.

Each session of Games 1 and 2 had six conditions with varying attentional load, *i.e.*, with varying number of objects on screen. In Game 1, the load-conditions were in 1, 1, 1, 2, 3, and 4 objects. The condition order was random in each game. In Game 2, the order was 1, 2, 2, 2, 3, and 4 objects. The durations of each of these load-conditions were 15 s, giving Games 1 and 2 total durations of 90 s. A total of 60 target events were presented in each game, 10 in each condition.

**Games 3 and 4** were multi-object tracking games like Games 1 and 2 and had an identical adaptation procedure so that Game 3 was adapted to foveal (one target) and Game 4 to peripheral (two targets) vision, like Games 1 and 2, respectively. In Games 3 and 4, however, the participants only had one- and two-object attentional loads. In two conditions, one or two objects were presented alone and the participant responded to target events like in Games 1 and 2. In two other conditions, the same one or two to-be-attended objects were presented with six concurrently moving distracter objects having a distinct complex shape. Also the distracters presented target-event-like shape changes that the participants were instructed not to respond to. Both target and distracter objects were 120 ± 36 pixels (1.53 ± 0.46 visual degrees) long, had ±30% hue variability, had movement dynamics identical to those in Games 1 and 2, and the same maximum of six false alarms. Each game thus comprised of four consecutive conditions, each lasting 30 s. During the total 2 min game duration, 40 target events (10 in each condition) were presented in the to-be-attended objects with a uniform inter-event onset-to-onset range of 1.5−4.5 s, mean 3.0 s.

**Game 5** was a continuous single-object tracking task where the subjects reported transient feature-change (as in Games 1−4), that started at a fixed salient change-magnitude level and decreased by a factor of 1.6 in 12 steps towards a magnitude of zero. *I.e.*, Game 5 did not adapt to the participant’s performance level. Each of the 12 steps lasted 12 s, giving a total theoretical game duration of 144 s. The game continued until in one consecutive set of 8 target events, the participant reported only 2 or less. The object length was 170 pixels (2.16 visual degrees) and it moved as the objects in Games 1−4. During the game, a total of 80 target events were presented with a uniform inter-event onset-to-onset range of 0.6−3.0 s, mean 1.8 s. A maximum of eight false alarms was allowed.

**Game 6** was a Go/No-Go 1-back working memory task where the subjects reported whether a given object was different from the previously presented object (*p* = 0.5) and withheld the response if it was the same regardless of object location (*p* = 0.5). Game 6 did not adapt to individual performance and yielded an accuracy of 0.80 ± 0.03 across subjects and weeks. As the memory load *per se* was negligible, this task predominantly tapped on the visual object representation functions. The objects were randomly picked from a pool of 15 distinct types, the same that presented in random locations of a 2 x 2 grid around a central fixation point (Figure 2). The objects were 150 ± 15 pixels (1.91 ± 0.20 visual degrees) long. The objects’ center-of-mass was locked to the center of the grid cell but during the presentation time of 1 ± 0.1 s, the object shape was variable as if it moved with dynamics identical to those in Games 1−5, *i.e.*, the rotation and curvature of the objects were randomly variable both during and between the presentations. The inter-stimulus-onset-to-onset interval was 2.5 ± 0.1 s and a total of 73 stimuli were presented, giving a total game duration of 183 s. A maximum of six false alarms was allowed per game.

**Game 7** was a threshold-stimulus-detection task^2–4^. Semi-transparent complex visual objects were presented randomly in the same visual field quadrants used in Game 6. The participant’s task was to report all perceived stimuli with a key press. The object transparency was adapted with data obtained from prior Game 7 games to the individual performance with an alpha parameter, *A*, so that a detection accuracy of 0.5 was obtained at 0.5*A*. During the games, unmoving images of the objects used in Games 1−6 were presented at a length of 120 ± 12 pixels (1.53 ± 0.15 visual degrees) for a duration of 0.1 s at five equiprobable levels of *A* so that *A* were 0, 0.25, 0.5, 0.75, and 1.0. The inter-stimulus-onset-to-onset interval was 2.0 ± 1.5 s and a total of 90 stimuli were presented, giving a total game duration of 180 s.

**SUPPLEMENTARY REFERENCES**

1. Rouhinen, S., Panula, J., Palva, J. M. & Palva, S. Load dependence of β and γ oscillations predicts individual capacity of visual attention. *J Neurosci*. **33,** 19023-19033 (2013).

2. Monto, S., Palva, S., Voipio, J. & Palva, J. M. Very slow EEG fluctuations predict the dynamics of stimulus detection and oscillation amplitudes in humans. *J Neurosci*. **28,** 8268-8272 (2008).

3. Palva, J. M. & Palva, S. Roles of multiscale brain activity fluctuations in shaping the variability and dynamics of psychophysical performance. *Prog Brain Res*. **193,** 335-350 (2011).

4. Palva, J. M. *et al.* Neuronal long-range temporal correlations and avalanche dynamics are correlated with behavioral scaling laws. *Proc Natl Acad Sci U S A*. **110,** 3585-3590 (2013).
